# HLA-DRB1 alleles and salivary *Streptococcus mutans* colonization in a group of Swedish children

**DOI:** 10.1186/s12903-025-06248-z

**Published:** 2025-05-27

**Authors:** Hanna Hänel, Dan Ericson, Marie Louise Wallengren

**Affiliations:** Department of Cariology, Faculty of Odontology, Malmö University, Malmö, Sweden

**Keywords:** HLA, *Streptococcus mutans*, Saliva, Caries, Genetics, DRB1*0401

## Abstract

**Background:**

Genetic factors significantly influence caries development and the colonization of oral bacteria, which could explain why some individuals are more prone or resistant to caries. Human leukocyte antigen (HLA) class II is a component in the adaptive immune system that has been associated with the colonization of oral bacteria such as *Streptococcus mutans*. This study aimed to investigate the association between specific alleles and genotypes of HLA-DRB1 on subgroup level and the colonization of *S. mutans* in a group of Swedish children.

**Methods:**

Blood samples from 357 children were analyzed for HLA using next generation sequencing. Saliva samples were collected and analyzed for *S. mutans*, after which the subjects were divided into three groups: low, moderate, and high levels of colony forming units (CFU). The frequency of DRB1 alleles and genotypes was compared between the three groups. In addition, colonization levels, including the extremely high and low *S. mutans* CFU in individuals with alleles DRB1*0401, *0404, and *0301 were compared to the rest of the material.

**Results:**

Individuals with DRB1*0401 were significantly associated with the extremely high CFU levels, since CFU levels > 100 were observed in 4.3% of individuals with DRB1*0401, compared to none among those without the allele (*p* = 0.009, Fisher’s exact test). No statistical association was noted between the low, moderate, and high *S. mutans* groups and specific alleles or genotypes.

**Conclusions:**

The findings suggest a potential relation between HLA class II alleles and the colonization of *S. mutans.* Specifically, carrying the DRB1*0401 allele may be a predisposing factor for higher levels of colonization.

## Background

Dental caries is the most common disease in the world, with approximately 2.5 billion people suffering from untreated caries in their primary and permanent dentition. The disease is multifactorial and complex, as well as expensive to treat for both individuals and society [1].

Caries is caused by plaque bacterial acids from sugar fermentation and is strongly affected by environmental factors such as diet, fluoride exposure, and socioeconomic position. Saliva in the oral environment also plays an important role in maintaining tooth integrity and interaction with microorganisms. In addition to these factors, research has shown that genetic host factors may play a significant role in caries development [2].

Already in the early 1950s, the Vipeholm study provided evidence that caries activity varies among individuals, even under uniform conditions [3]. This variation has been attributed to a biological, genetic component that makes some individuals more prone or resistant to caries [4]. Several studies have since then established the role of heredity in the development of caries. For instance, in monozygotic twins raised apart and exposed to different environmental factors, genetic factors could explain around half of the variance for the number of teeth/surfaces restored and caries present [5]. In 20,000 Swedish twin pairs, genetic factors could explain more than half of the variations in caries scores and caries trajectories [6]. A continued mapping of the relationship between genetics and caries is important in order to explain the mechanism behind the most common non communicable disease.

### Mutans streptococci

No single bacteria species is solely responsible for caries, but acidogenic bacteria—such as mutans streptococci (MS), including *Streptococcus mutans*—are a significant contributor because of their ability to thrive in an acidic environment [7]. Since caries is a multifactorial disease, colonization by a specific species will not be the only factor that determines disease development, but elevated levels of MS are consequently associated with caries disease in both adults and children [8–10].

Variations in MS colonization are also affected by genetic host factors, and Corby et al. estimated the genetic component to be 52% [11]. Esberg et al. also showed that the composition of oral microbiota and the presence and quantity of *S. mutans* were strongly correlated with host genetic factors [12].

### Human leukocyte antigen

One host genetic factor influencing the colonization of microorganisms is a set of genes in the human leukocyte antigen (HLA) class II gene complex. One function of HLA class II is to bind peptides from non-self antigens and present them to T cells, a process that initiates the adaptive immune response. Every human carries two alleles of each HLA gene, and these genes are highly polymorphic and diverse in a population, which can explain the differences in antigen recognition capacity and immunological responses among individuals. Accordingly, HLA has been investigated in relation to both autoimmune and infectious diseases [13, 14]. Research has found lower T-cell activity against purified streptococcal antigen in caries prone individuals [15], as well as a reduced T-cell expression in children with early childhood caries [16].

### Research on HLA, MS, and caries

Already in 1981, Lehner et al. suggested a correlation between HLA-DR alleles and caries and reported a significant association between HLA-DR4 and caries-prone individuals [15]. Since then, research on HLA-DRB1 and caries has had contradictory results—showing associations between HLA-DRB1*04 and caries [17, 18], associations to other DRB1-alleles [18, 19], or no associations at all [20–22].

Our study group is interested in specific individual immunological factors that can affect the ability of the adaptive immune response to oral bacteria; therefore, we found it relevant to investigate the possibility that different HLA-DRB1 profiles affect the colonization of *S. mutans.* We showed a relationship between HLA-DR4 and MS, with a higher colonization grade in HLA-DR4 positive renal transplant subjects [23]. Further research showed lower salivary immunoglobulin A (IgA) activity to *S. mutans* in DRB1*04 positive individuals, particularly in the subgroups DBR1*0401 and *0404 [24, 25]. It implies that the salivary IgA response in certain HLA-DRB1 subgroups might be lower, which could facilitate an increased oral *S. mutans* colonization [26].

### Objectives and aims

Motivated by our previous results in adults [23, 27] we hypothesized that there exists an association between specific alleles of HLA-DRB1 and colonization of *S. mutans*. In this study we focused on children since MS are usually established in the oral cavity at an early age. Identifying factors associated with colonization of MS is important, as both the colonization itself and its timing affects caries risk [28].

We aimed to investigate associations between *S. mutans* and HLA-DRB1 alleles on a subgroup level. Some HLA alleles may promote resistance to disease, which is the case in, for example, type 1 diabetes [29]. Therefore, to minimize the risk of alleles masking other alleles promoting susceptibility to colonization, we also investigated DRB1 genotypes.

## Material and methods

This article reports on the Mutans Prediction in Skåne (MuPiS) project. The overall aim of the MuPiS project is to study the HLA profile of the host in relation to the colonization of *S. mutans*. In this study, we analyzed the association between HLA-DRB1 alleles and genotypes, and CFU levels of *S. mutans* colonization in children. The MuPiS project was approved by the Local Ethics Committee at Lund University (Dnr 536/2006).

### Study population

The children in the MuPiS project are recruited from another comprehensive project, Diabetes Prediction in Skåne (DiPiS) [30]. A total of 1620 children, aged 5–8 years, were invited to participate in the MuPiS project and mothers of 909 children gave their informed written consent to participate. The children were divided into two groups according to HLA genotypes. Those expected to carry HLA-DRB1*04 (*n* = 527) and those who did not (*n* = 382). Complete data was missing from 552 children, and they were therefore excluded from this analysis, resulting in a final study group of 357 children.

### HLA typing

Blood samples were sent to Scisco Genetics Inc. laboratory in the USA for (additional) HLA typing, performed with HLA next generation sequencing (NGS) analysis as described by Smith et al. [31]. Due to sampling and storing problems as well as loss in HLA NGS analysis, we excluded an additional 52 children from the study group, so the study finally included 305 children. The gender distribution was even with 153 girls and 152 boys.

### Bacterial sample collection and cultivation

The subjects were asked to chew paraffin while saliva was collected. The saliva samples were then immediately stored in − 18 °C for up to three months maximum. Thereafter, the samples were stored in a freezer in − 80˚C until processing. The method as well as the media used for cultivating MS is in accordance with the modified principles developed by Gold et al. [32]. Before cultivation, the saliva Eppendorf tubes were thawed in cold water for five minutes and mixed carefully. Subsequently, 100 µl of undiluted saliva from each sample were spread on sterile mitis salivarius bacitracin agar plates by using glass beads (5 mm). The mitis salivarius bacitracin medium contained 0.2 units of bacitracin/ml and 15% sucrose. All plates were incubated at 37 °C for 72 h under anaerobic conditions (5% CO_2_ and 95% N_2_). Colony forming units (CFU) representing morphological types of *S. mutans* were counted using a microscope, according to the colony morphology identification method described by Villhauer et al. [33]. All laboratory procedures were performed by the same medical laboratory engineer with a long experience in colony identification.

### Statistical analysis

Using the chi-square test, the frequencies of allele genotypes were compared between three groups of salivary *S. mutans*: low, moderate, and high levels of CFU. Colonization of *S. mutans* in individuals with the three most common and interesting alleles—namely DRB1*0401, *0404, and *0301 [22, 24, 25]—were compared to the rest of the material using T-test and linear scale. Extremely low and extremely high colonization grade of *S. mutans* in the material was compared between individuals with alleles DRB1*0401, *0404, and *0301, respectively, and the rest of the material using Fisher’s exact test. *P* values less than 0.05 were considered to indicate statistical significance.

## Results

### Frequency of HLA-DRB1 alleles

Altogether, 25 different HLA-DRB1 alleles occurred in the material (Table 1). A total of 273 children were dizygotic for the DRB1 allele, while 32 children were homozygotic. Genotype frequencies are presented in Table 2.

**Table 1:** Frequency of HLA-DRB1 alleles

HLA-DRB1 allele	Allele frequency (n)
0101	50
0102	3
0103	1
0301	150
0401	149
0402	8
0403	11
0404	69
0405	5
0407	2
0701	40
0801	22
0901	8
1001	5
1101	6
1104	2
1201	3
1301	3
1302	44
1303	1
1401	12
1501	6
1502	4
1601	5
1602	1
Total	610

**Table 2:** Frequency of HLA-DRB1 genotypes

HLA-DRB1 genotype	Genotype frequency (n)
0101/0301	8
0101/0401	26
0101/0404	8
0301/0301	17
0301/0401	41
0301/0404	17
0301/1302	14
0401/0401	8
0401/0404	9
0401/0701	7
0401/0801	8
0401/1302	15
0404/0701	8
0404/1302	8
Other	111
Total	305

### Colonization of *S. mutans*

*S. mutans* CFU varied between 0 and 600. Three groups of *S. mutans* CFU were made based on frequencies in the material: 70 subjects with CFU 0–9 were assigned to the low group, 174 subjects with CFU 10–49 to the moderate group, and 61 with CFU ≥ 50 to the high group (Table 3). In addition, colonization levels, including the extremely high and low *S. mutans* CFU, in individuals with alleles DRB1*0401, *0404, and *0301 were compared to the rest of the material. Ten subjects had extremely low values (CFU = 0) and six individuals had extremely high values (CFU > 100).

**Table 3:** Frequency of *S. mutans *CFU, divided into three groups

Group	CFU	Individuals (n)
Low, *n* = 70	0	10
	2	14
	3	6
	4	2
	5	35
	8	3
Moderate, *n* = 174	10	66
	15	13
	20	48
	30	34
	40	13
High, *n* = 61	50	29
	60	6
	70	4
	80	2
	100	14
	120	1
	130	1
	240	1
	500	2
	600	1
	Total	305

### Colonization of *S. mutans* and prevalence of HLA-DRB1 alleles

**Fig. 1:**
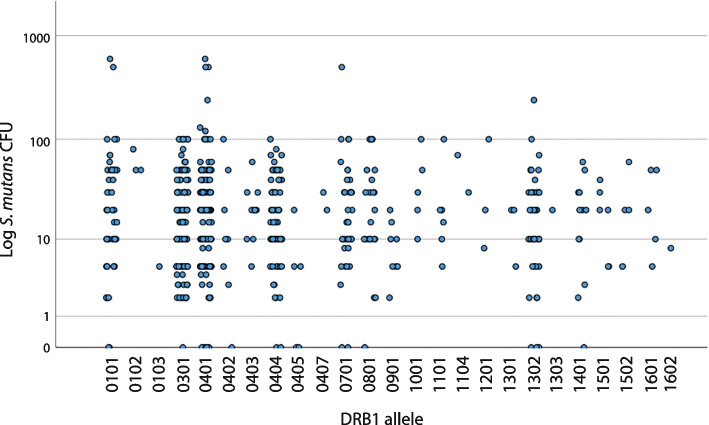
A scatter plot illustrating the distribution of *S. mutans* colonization (CFU) across all HLA-DRB1 alleles (*n* = 578) in the material (305 individuals). Homozygotic individuals (*n* = 32) are only included once. No clear associations between *S. mutans* colonization and certain alleles can be observed

Figure 1 shows all occurring alleles in the material and the distribution of *S. mutans* CFU. A relatively even distribution was noted for the different alleles. No significant association between the three *S. mutans* groups and specific alleles was seen.

### Colonization of *S. mutans* and prevalence of HLA-DRB1 genotypes

**Fig. 2:**
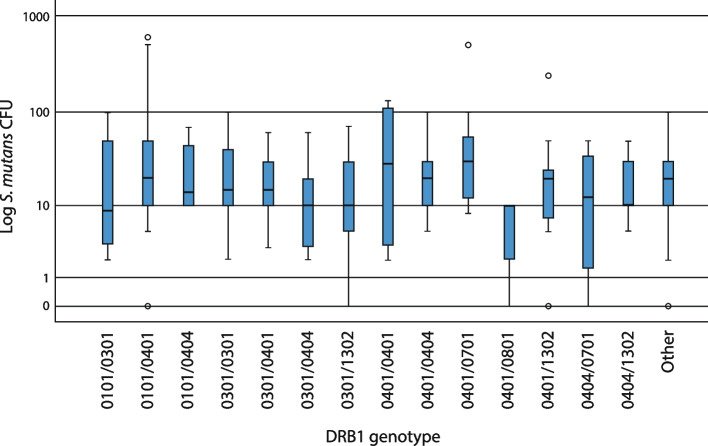
A box plot illustrating the distribution of *S. mutans* colonization (CFU) across HLA-DRB1 genotypes occurring in seven or more subjects (194 subjects included). A total of 111 subjects with less common HLA-DRB1 genotypes constitute the group “Other”. No significant associations were observed

Figure 2 shows the most frequent genotypes, namely those occurring in seven or more subjects, including 194 subjects. A total of 111 subjects with less common genotypes constitute the group “Other.” No significant association between the three *S. mutans* groups and specific genotypes was seen, although a tendency to have higher median levels was noted for DRB1*0401/0701. None of the eight subjects with DRB1*0401/0801 had CFU > 10, and none of the eight subjects with DRB1*0101/0404 had CFU < 10. Allelic homozygosity (represented by DRB1*0301/0301 and *0401/0401) did not significantly affect the degree of colonization either, although a tendency to have higher median values was seen in genotypes DRB1*0401/0401.

### Colonization of *S. mutans* in individuals with the most occurring alleles

**Fig. 3:**
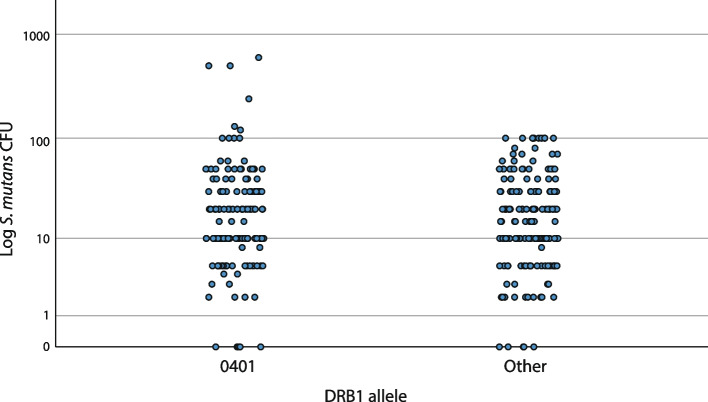
A scatter plot of *S. mutans* colonization (CFU) and presence of allele DRB1*0401 (*n* = 141) compared to non-DRB1*0401 (Other) (*n* = 164). CFU levels > 100 were observed in 4.3% of individuals with DRB1*0401, compared to 0% among those without the allele (*p* = 0.009, Fisher’s exact test)

**Fig. 4:**
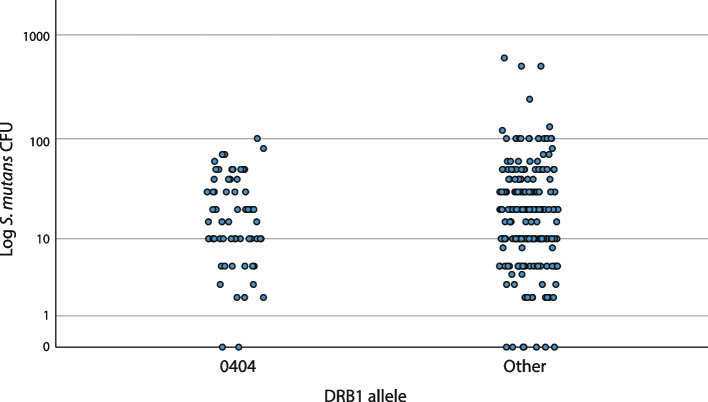
A scatter plot of *S. mutans* colonization (CFU) and presence of allele DRB1*0404 (*n* = 65) compared to non-DRB1*0404 (Other) (*n* = 240). No significant associations were observed

**Fig. 5:**
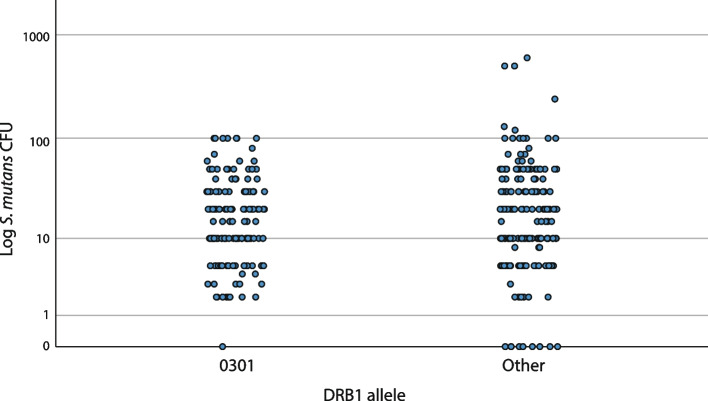
A scatter plot of *S. mutans* colonization (CFU) and presence of allele DRB1*0301 (*n* = 133) compared to non-DRB1*0301 (Other) (*n* = 172). No individuals with DRB1*0301 had CFU values > 100 and only one had CFU = 0

Figures 3, 4, and 5 show the colonization of *S. mutans* in the three most occurring and interesting alleles in the material—DRB1*0401, *0404, and *0301—in comparison with the rest of the material. Individuals with DRB1*0401 were significantly associated with extremely high CFU levels since CFU levels > 100 were observed in 4.3% of individuals with DRB1*0401, compared to none among those without the allele (*p* = 0.009, Fisher’s exact test) (Fig. 3). Additionally, individuals with DRB1*0301 had a significantly lower CFU mean value compared to others (*p* = 0.037, t-test), but only one individual with DRB1*0301 had an extremely low CFU value (Fig. 5). There was no significant association between any specific alleles and extremely low CFU levels. No gender differences were found in the results (not shown).

## Discussion

Genome-wide analyses of dental caries have identified multiple loci for dental caries, including haplotypes at the HLA region of chromosome 6 [34, 35]. These studies are not driven by a specific hypothesis. In contrast, the present study is hypothesis driven and based on the association of HLA with antigen recognition and immune response. Initiated by results from previous studies by our study group, we hypothesized that there exists an association between specific alleles of HLA-DRB1 and the colonization of *S. mutans*, namely that certain HLA-DRB1 alleles are not as immunologically responsive to *S. mutans* as others, thereby demonstrating higher colonization levels [23–25].

Our results established a statistically significant association between DRB1*0401 and the extremely high colonization level of *S. mutans* (Fig. 3). More individuals with higher CFU values were seen for DRB1*0401 compared to other alleles. DRB1*0401 was present in half of the individuals with CFU ≥ 100. Furthermore, all six individuals with the highest colonization (CFU > 100) had DRB1*0401 as one or both of their alleles (Fig. 3). This is in line with previous results by our study group [24, 25], and other study groups have reported comparable findings [22].

Our previous results indicated that individuals with DRB1*0401 or *0404 have a lower salivary IgA reaction to *S. mutans* [24, 25]. It is an interesting finding because previous research shows the specific immune response of the individual against mutans streptococci is provided mainly by IgA [26].

Acton et al. found significant associations between colonization of *S. mutans* and DRB1*3 [22]. We could not confirm this association in our material, nor could Altun et al. [36]. Instead, we found that DRB1*0301 had a significantly lower CFU mean value and the allele was not associated with either extremely high or low CFU levels (Fig. 5).

When analyzing genotypes, we found no statistically significant associations between any DRB1 genotypes and *S. mutans* (Fig. 2). Although we could see an interesting tendency to have higher median colonization values in DRB1*0401/0701 (*n* = 7) and *0401/0401 (*n* = 8) genotypes. Both genotypes contain DRB1*0401, which is in line with our findings that DRB1*0401 is associated with higher *S. mutans* CFU.

Furthermore, none of the eight subjects with DRB1*0401/0801 had CFU > 10. Since some HLA alleles may promote resistance to disease [29], it might be that DRB1*0801 promotes resistance to colonization.

### Study strengths and limitations

This study includes a uniquely large sample in comparison with other studies on the same topic. The only previous similar study assessing this association in children was limited by a rather small sample size [36]. We here present a sample of 357 (305 analyzed) healthy children with access to regular free dental visits and prophylaxis. The children represent a relatively homogenous Swedish population, which facilitates comparison within the group, but generalizing to other populations should be done considering variations in genomes. Also, mothers who consented to their children’s participation may represent a more socioeconomically stable group, potentially influencing the children’s caries risk factors, among them *S. mutans* colonization. [37].

We analyzed the association between *S. mutans* and part of the host HLA-DRB1 gene profile. Both individual alleles on a four-digit subgroup level and certain genotypes within HLA-DRB1 were studied. Besides the comparison between HLA-DRB1 and *S. mutans* colonization it would have been preferable with a multivariate analysis, since confounding factors such as caries status, oral hygiene and diet also influence the colonization [38]. Multivariate analysis was not feasible in this study due to lack of information on other variables.

Furthermore, the identification of *S. mutans* colonies was based on MSB agar morphology. The use of a more precise identification method might have enhanced the reliability of the results [33]. However, the colonization prevalence of *S. mutans* in children has been reported as significantly higher compared to *S. sobrinus*, which reduces the risk of misidentification [39].

### Implications for future research

All these studies with contradictory results amply illustrate the difficulties in studying the involvement of genetic factors for multifactorial diseases such as caries and the colonization of *S. mutans*. The inherent genetic component of caries and oral bacterial colonization is likely attributed not to a single HLA allele but rather to patterns of HLA polymorphism as well as interactions with other genes and environmental factors [2, 40, 41]. Therefore, it is important to consider that the nature of caries, as well as colonization, prevents the conclusion that the presence of a single HLA allele is sufficient for the outcome of disease. By continuing to investigate the genetic component in caries, we might get a more precise understanding of interindividual differences in the caries progression and colonization of oral bacteria. Further studies are needed to elucidate the influence of host genes such as HLA on the microbial colonization of the oral cavity.

## Conclusion

The findings in this study suggest a potential relation between HLA class II alleles and the colonization of *S. mutans.* Specifically, carrying the DRB1*0401 allele may be a predisposing factor for higher levels of colonization. This information could be relevant for caries risk assessment and individual preventive measures.

## Data Availability

The datasets used and/or analyzed during the current study are available from the corresponding author on reasonable request.
